# Complete Genome Sequence of Salmonella enterica Myophage Matapan

**DOI:** 10.1128/MRA.01017-19

**Published:** 2019-09-19

**Authors:** Jasmine Juliette, Yicheng Xie, Heather Newkirk, Mei Liu, Jason J. Gill, Jolene Ramsey

**Affiliations:** a Center for Phage Technology, Texas A&M University, College Station, Texas, USA; Queens College

## Abstract

Here, we describe the Salmonella enterica serovar Heidelberg phage Matapan. A myophage with a 157,408-kb genome, Matapan is most closely related to Vi01-like phages.

## ANNOUNCEMENT

Salmonella enterica serovar Heidelberg is a member of Enterobacteriaceae and a zoonotic pathogen (1). Strains in this serovar cause human salmonellosis, are multidrug resistant, and are detectable in poultry and other meat processing plants across the United States (1, 2). Salmonellosis is spread through infected animal products, feces, contaminated water, and human-to-human contact. Using bacteriophage therapy to reduce *S.* Heidelberg transmission among animals during transport and during processing is desired to lower the number of human infections (3). Here, we present the genome sequence of Matapan, a myophage that infects *S*. Heidelberg.

Matapan was collected from filtered (0.2-μm pore size) municipal wastewater in College Station, TX, via enrichment on *S.* Heidelberg. Host bacteria were cultured on tryptic soy broth or agar (Difco) at 37°C with aeration, and phage isolation and propagation were done using the soft agar overlay method (4). Phage morphology was determined by negatively staining samples with 2% (wt/vol) uranyl acetate and imaging by transmission electron microscopy at the Texas A&M Microscopy and Imaging Center (5). The genomic DNA for Matapan was purified using the Promega Wizard DNA clean-up system, according to the modification in the shotgun library preparation protocol given by Summer (6). Libraries were prepared with an Illumina TruSeq Nano low-throughput kit, according to the manufacturer's recommendations. Sequencing was performed on an Illumina MiSeq instrument with v2 500-cycle chemistry in a paired-end, 250-bp run, yielding 1,167,981 total reads. Sequence reads were quality controlled with FastQC (http://www.bioinformatics.babraham.ac.uk/projects/fastqc/) and trimmed with the FASTX-Toolkit 0.0.14 (http://hannonlab.cshl.edu/fastx_toolkit/). The genome was assembled to 26.9-fold coverage with SPAdes v3.5.0, with default parameters, and closed by PCR (forward primer, 5′-GCCACAGAGAACCCATGAA-3′; reverse primer, 5′-ATAAACCTGAATGTCCAGACCA-3′) and Sanger sequencing (7). The analysis and annotation tools are hosted on the Center for Phage Technology Galaxy and Web Apollo instances (https://cpt.tamu.edu/galaxy-pub/) (8, 9). Genes were predicted using Glimmer v3.0 and MetaGeneAnnotator v1.0 and ARAGORN v2.36 for tRNAs (10–12). The presence of Rho-independent terminators was predicted with TransTermHP v2.09 (13). Predicted protein functions were assigned using InterProScan v5.22-61 and BLAST v2.2.31 versus NCBI nonredundant (nr), UniProtKB Swiss-Prot, and TrEMBL databases, with a 0.001 maximum expectation cutoff (14–16). Whole-genome sequence identities were calculated with progressiveMauve v2.4.0 (17).

Matapan is a Vi01-like myophage with a 157,408-kb genome with a G+C content of 44.9%, a 93.0% coding density, 214 protein-coding genes, and 3 tRNAs. As determined by progressiveMauve, Matapan shares nucleotide similarities of 91.3%, 89.5%, 88.2%, and 87.9% with *Salmonella* phage Vi01 (GenBank accession number FQ312032), *Escherichia* phage ECML-4 (GenBank accession number JX128257), *Salmonella* phage STML-13-1 (GenBank accession number JX181828), and *Salmonella* phage Marshall (GenBank accession number KF669653), respectively, and 196 similar proteins with phage Vi01. Similar to other Vi01-like phages, Matapan is predicted to use headful or pac-type packaging using PhageTerm (18). About one-third of the predicted proteins were assigned putative functions. Similarities to phage T4 in the structural region led to reopening the genome according to convention, in front of the *rIIA*-like gene.

### Data availability.

The genome sequence and associated data for phage Matapan were deposited under GenBank accession number MN066127, BioProject accession number PRJNA222858, SRA accession number SRR8869240, and BioSample accession number SAMN11360418.

## References

[1] Rothrock MJ Jr, Ingram KD, Gamble J, Guard J, Cicconi-Hogan KM, Hinton A, Hiett KL 2015 The characterization of Salmonella enterica serotypes isolated from the scalder tank water of a commercial poultry processing plant: recovery of a multidrug-resistant Heidelberg strain. Poult Sci 94:467–472. doi:10.3382/ps/peu060. 25681479

[2] Vincent C, Usongo V, Berry C, Tremblay DM, Moineau S, Yousfi K, Doualla-Bell F, Fournier E, Nadon C, Goodridge L, Bekal S 2018 Comparison of advanced whole genome sequence-based methods to distinguish strains of Salmonella enterica serovar Heidelberg involved in foodborne outbreaks in Québec. Food Microbiol 73:99–110. doi:10.1016/j.fm.2018.01.004. 29526232

[3] Wall SK, Zhang J, Rostagno MH, Ebner PD 2010 Phage therapy to reduce preprocessing Salmonella infections in market-weight swine. Appl Environ Microbiol 76:48–53. doi:10.1128/AEM.00785-09. 19854929 PMC2798657

[4] Adams MH 1956 Bacteriophages. Interscience Publishers, Inc., New York, NY.

[5] Valentine RC, Shapiro BM, Stadtman ER 1968 Regulation of glutamine synthetase. XII. Electron microscopy of the enzyme from Escherichia coli. Biochemistry 7:2143–2152. doi:10.1021/bi00846a017. 4873173

[6] Summer EJ 2009 Preparation of a phage DNA fragment library for whole genome shotgun sequencing. Methods Mol Biol 502:27–46. doi:10.1007/978-1-60327-565-1_4. 19082550

[7] Bankevich A, Nurk S, Antipov D, Gurevich AA, Dvorkin M, Kulikov AS, Lesin VM, Nikolenko SI, Pham S, Prjibelski AD, Pyshkin AV, Sirotkin AV, Vyahhi N, Tesler G, Alekseyev MA, Pevzner PA 2012 SPAdes: a new genome assembly algorithm and its applications to single-cell sequencing. J Comput Biol 19:455–477. doi:10.1089/cmb.2012.0021. 22506599 PMC3342519

[8] Afgan E, Baker D, Batut B, van den Beek M, Bouvier D, Cech M, Chilton J, Clements D, Coraor N, Grüning BA, Guerler A, Hillman-Jackson J, Hiltemann S, Jalili V, Rasche H, Soranzo N, Goecks J, Taylor J, Nekrutenko A, Blankenberg D 2018 The Galaxy platform for accessible, reproducible and collaborative biomedical analyses: 2018 update. Nucleic Acids Res 46:W537–W544. doi:10.1093/nar/gky379. 29790989 PMC6030816

[9] Lee E, Helt GA, Reese JT, Munoz-Torres MC, Childers CP, Buels RM, Stein L, Holmes IH, Elsik CG, Lewis SE 2013 Web Apollo: a Web-based genomic annotation editing platform. Genome Biol 14:R93. doi:10.1186/gb-2013-14-8-r93. 24000942 PMC4053811

[10] Delcher AL, Harmon D, Kasif S, White O, Salzberg SL 1999 Improved microbial gene identification with GLIMMER. Nucleic Acids Res 27:4636–4641. doi:10.1093/nar/27.23.4636. 10556321 PMC148753

[11] Noguchi H, Taniguchi T, Itoh T 2008 MetaGeneAnnotator: detecting species-specific patterns of ribosomal binding site for precise gene prediction in anonymous prokaryotic and phage genomes. DNA Res 15:387–396. doi:10.1093/dnares/dsn027. 18940874 PMC2608843

[12] Laslett D, Canback B 2004 ARAGORN, a program to detect tRNA genes and tmRNA genes in nucleotide sequences. Nucleic Acids Res 32:11–16. doi:10.1093/nar/gkh152. 14704338 PMC373265

[13] Kingsford CL, Ayanbule K, Salzberg SL 2007 Rapid, accurate, computational discovery of Rho-independent transcription terminators illuminates their relationship to DNA uptake. Genome Biol 8:R22. doi:10.1186/gb-2007-8-2-r22. 17313685 PMC1852404

[14] Jones P, Binns D, Chang H-Y, Fraser M, Li W, McAnulla C, McWilliam H, Maslen J, Mitchell A, Nuka G, Pesseat S, Quinn AF, Sangrador-Vegas A, Scheremetjew M, Yong S-Y, Lopez R, Hunter S 2014 InterProScan 5: genome-scale protein function classification. Bioinformatics 30:1236–1240. doi:10.1093/bioinformatics/btu031. 24451626 PMC3998142

[15] Camacho C, Coulouris G, Avagyan V, Ma N, Papadopoulos J, Bealer K, Madden TL 2009 BLAST+: architecture and applications. BMC Bioinformatics 10:421. doi:10.1186/1471-2105-10-421. 20003500 PMC2803857

[16] UniProt Consortium. 2019 UniProt: a worldwide hub of protein knowledge. Nucleic Acids Res 47:D506–D515. doi:10.1093/nar/gky1049. 30395287 PMC6323992

[17] Darling AE, Mau B, Perna NT 2010 progressiveMauve: multiple genome alignment with gene gain, loss and rearrangement. PLoS One 5:e11147. doi:10.1371/journal.pone.0011147. 20593022 PMC2892488

[18] Garneau JR, Depardieu F, Fortier L-C, Bikard D, Monot M 2017 PhageTerm: a tool for fast and accurate determination of phage termini and packaging mechanism using next-generation sequencing data. Sci Rep 7:8292. doi:10.1038/s41598-017-07910-5. 28811656 PMC5557969

